# Novel compound heterozygous mutations of *DNAH5* identified in a pediatric patient with Kartagener syndrome: case report and literature review

**DOI:** 10.1186/s12890-021-01586-4

**Published:** 2021-08-14

**Authors:** Lina Wang, Xin Zhao, Hang Liang, Li Zhang, Chunyan Li, Deli Li, Xiangfeng Meng, Fanzheng Meng, Mao Gao

**Affiliations:** 1 Pediatric Department of Respiration II, The First Hospital of Jilin University, No.71 Xinmin Street, Changchun, 130000 China; 2 Jilin University, Changchun, China

**Keywords:** Bronchoscopy, Children, DNAH5, Kartagener syndrome, KS, Whole-exome sequencing

## Abstract

**Background:**

Kartagener syndrome is a subtype of primary ciliary dyskinesia that may exhibit various symptoms including neonatal respiratory distress and frequent infections of the lung, sinus and middle ear because of the impaired function of motile cilia. In addition to typical symptoms of primary ciliary dyskinesia, patients with Kartagener syndrome also show situs inversus. It is an autosomal recessive disorder which is mostly caused by mutations in *DNAH5*. Kartagener syndrome is often underdiagnosed due to challenges in the diagnosis process. As next-generation sequencing becomes widely used in clinical laboratories, genetic testing provides an accurate approach to the diagnosis of Kartagener syndrome.

**Case presentation:**

A 7-year-old female patient presented with runny nose of 6 years duration and recurrent cough with phlegm of 2 years duration. Kartagener syndrome was diagnosed through diagnostic tests such as nasal nitric oxide (NO) concentration and transmission electron microscopy, and after performing other exams that corroborated the diagnosis, such as computed tomography, bronchoscopy and hearing test. Whole-exome sequencing was performed for the patient and both parents. The pediatric patient was diagnosed as Kartagener syndrome with the typical symptoms of ciliary dyskinesia including bronchiectasis, sinusitis, conductive hearing loss and situs inversus along with a reduced nasal NO concentration and ciliary abnormalities. The patient carried two novel compound heterozygous mutations in *DNAH5*, NM_001369:c.12813G > A (p. Trp4271Term) and NM_001369:c.9365delT (p. Leu3122Term). Both mutations lead to premature stop codons and thus are pathogenic. The p. Trp4271Term and p. Leu3122Term mutations were inherited from the father and the mother of the patient individually. A literature review was also conducted to summarize *DNAH5* mutations in pediatric patients with Kartagener syndrome across different ethnic groups.

**Conclusions:**

Our study provides a good example of the diagnosis of Kartagener syndrome in pediatric patients using a series of diagnostic tests combined with genetic testing. Two novel loss-of-function mutations in *DNAH5* were identified and validated in a pediatric patient with Kartagener syndrome.

**Supplementary Information:**

The online version contains supplementary material available at 10.1186/s12890-021-01586-4.

## Background

Kartagener syndrome (KS) is a rare autosomal recessive genetic disease. It is a subtype of primary ciliary dyskinesia (PCD), characterized by situs inversus accompanying the typical PCD symptoms. KS often starts in early childhood with chronic respiratory symptoms that are non-specific, making it normally undiagnosed. The presence of the clinical triad of bronchiectasis, sinusitis and situs inversus is currently used as the standard for diagnosis of KS. Nasal NO concentration and pathological diagnosis through transmission electron microscopy (TEM) analysis of ciliary ultrastructure and high speed video microscopy analysis (HSVMA) of cilia beat pattern with live tissue are used for the diagnosis of KS [1]. In addition, genetic testing is becoming more common in the diagnosis of KS in the past two decades due to recent advances in next-generation sequencing, which serves as an accurate diagnostic approach. Early diagnosis and treatment of KS are critical for increasing the quality of life and survival time of pediatric patients. In the current study, we report a Chinese pediatric patient with KS caused by two compound heterozygous mutations in *DNAH5*. We present the clinical manifestations and diagnostic processes of the patient as well as the identification of two novel pathogenic loss-of-function *DNAH5* mutations.

## Case presentation

A 7-year-old female was diagnosed as pneumonia in a local hospital for having wet cough for twenty days without improvement in her condition. Her chest X-ray revealed dextrocardia and she was then transferred to our hospital. The patient had a history of a runny nose for 6 years and intermittent attacks of wet cough for 2 years. The cough usually got worse after a common cold or flu, accompanied by yellow phlegm, and was relieved after symptomatic treatment. The patient was born at term without a respiratory distress and kept healthy before the age of one. She was the only child of the family and refused the familial history of the condition. The physical examination showed tenderness in the sinus area, pharyngeal congestion and coarse crackles in both lungs. Her apical beat was located at the fifth intercostal space at the right midclavicular line with normal heart rhythm and a heart rate of 102 beats per minute. No pathological murmurs and extra heart sounds were detected. Her abdominal examination was normal. She was admitted with a diagnosis of pneumonia. At the follow-up, the patient did not have a fever but still had chronic cough with phlegm, which could be temporarily relieved by the acetylcysteine solution for inhalation treatment, and a mild runny nose. The patient currently has normal growth and development.

Additional examinations were performed as follows.

**Fig. 1:**
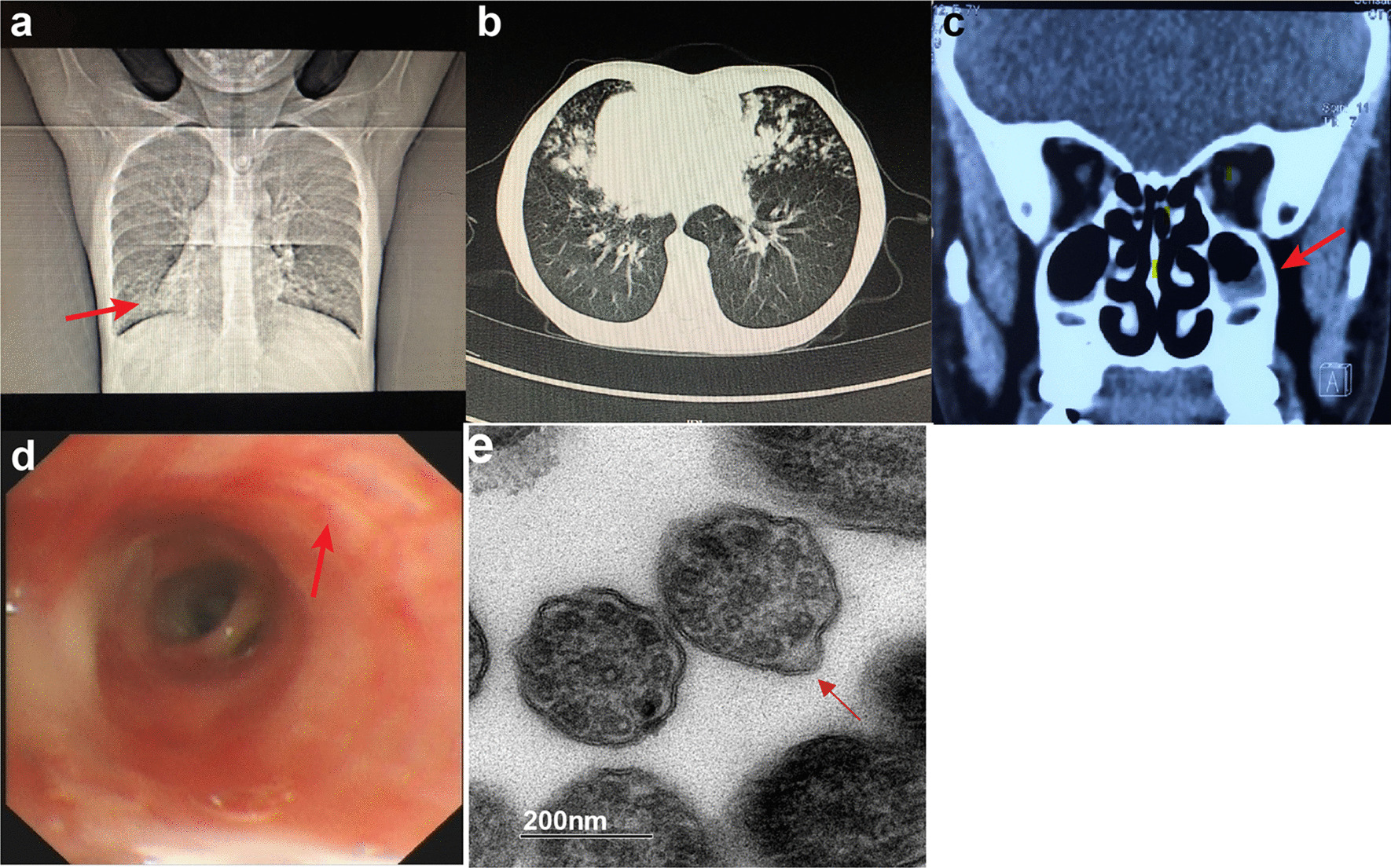
Image test, bronchoscopy and TEM results. **a** chest X-ray shows dextrocardia and bronchial inflammation. Arrow indicates the apex of the heart. **b** Chest CT shows dextrocardia, bronchiectasis and mucus plugs. **c** Sinus CT shows rhinosinusitis. Arrow indicates mucosal thickening in the maxillary sinuses. **d** bronchoscopy shows thinned mucosa, sharpened tracheal ring and a large amount of white sputum. Arrow indicates a white nodular protuberance, which is caused by mucus thinning accompanied by bulging of cartilage rings. **e** TEM of biopsies from bronchial mucosa shows wrinkled surfaces and lost integrity of cilia. Microtubular disorganization, partial loss of outer dynein arms and reduction of outer dynein arms are detected. Arrow indicates an axoneme with absence of outer dynein arms (TEM images resolution: 2048 × 2048 pixels without any downstream processing)
- *Imaging tests* Chest X-ray and chest computed tomography (CT) revealed bilateral pneumonia with potential bronchiectasis and mucus plugs in the middle lobe and the lingula of the lung (Fig. 1a, b). Situs inversus was also revealed by the chest X-ray and chest CT and further confirmed by echocardiogram. Sinus CT revealed mucosal thickening in maxillary sinuses and ethmoid sinuses, and soft tissue high-density shadows in several sinus cavities, suggesting rhinosinusitis (Fig. 1c).
- *Bronchoscopy* Results of the bronchoscopy indicated endobronchial inflammation and bronchiectasis in the right middle lobe and the lingula of the left lung (Fig. 1d). The tracheal mucosa was smooth. A sharply demarcated carina was observed with inversely positioned bronchial structures. The bronchial mucosa of the upper lobe of the left lung was smooth, but all segments of the left lung had thinned mucosa and sharpened tracheal ring. All segments of the right lung had smooth bronchial mucosa but exhibited thinned mucosa and white nodular protuberances, which was caused by mucus thinning accompanied by bulging of cartilage rings. A large amount of white sputum was present in all levels of the tracheobronchial tree, which was cleared by bronchoscopic sputum suction. Bronchoalveolar lavage (BAL) was performed for the B4 and B5 segments of the left lung and B4, B5 and B8 segments of the right lung. The BAL fluid was turbid and positive for Haemophilus influenzae (Hin).
- *Hearing tests* The pure-tone audiometry results demonstrated conductive hearing loss. The air-bone gaps were 20 dB for both the left and right ears. Tympanometry resulted in a type B tympanogram, and the acoustic reflex was absent.
- *Pulmonary function tests* Pulmonary function tests indicated mild restrictive and obstructive ventilatory defects. And the forced expiratory volume in 1 s (FEV1) was improved by 24% after bronchodilator.
- *Laboratory tests* Complete blood count (CBC) and the value of procalcitonin (PCT) and C-reactive protein (CRP) were normal. The patient was positive for IgM antibodies to Mycoplasma pneumoniae while negative for all other pathogens tested. Analysis of lymphocyte subsets (TBNK panel) showed reduced level of Helper T cells [CD4 + 22.4% (29.0–45.0%)] and B cells [CD19 + 13.1% (18.0–28.0%)]. Other laboratory tests were normal.
- *Genetic tests* Whole-exome sequencing of the patient identified two novel compound heterozygous mutations, NM_001369:c.12813G > A (p. Trp4271Term) and NM_001369:c.9365delT (p. Leu3122Term), in the *DNAH5* gene (Fig. 2). These nonsense single nucleotide variant and frameshift deletion both result in premature stop codons. These two mutations and their inheritance were confirmed by Sanger sequencing.

- *Nasal NO concentration* The nasal NO concentration was 6 parts per billion (ppb), suggesting a possibility of KS, PCD, cystic fibrosis, severe rhinosinusitis, and severe nasal polyp.
- *TEM* TEM images were taken using the FEI Tecnai G2 Spirit BioTwin transmission electron microscope (Model No. 943205018411, FEI Company, Czech Republic) together with the Gatan CCD camera. Biopsies from bronchial mucosa demonstrated abnormal ciliary ultrastructures. Cilia showed wrinkled surfaces and lost integrity. Abnormal arrangement and number of microtubules were detected. Some of the microtubules had partial loss of outer dynein arms, reduction of outer dynein arms and immature cilia with internal disorganization. In addition, many cilia were fused into gigantic cilia with unclear axonemal structures (Fig. 1e and Additional file 2: Figure S2).

**Fig. 2:**
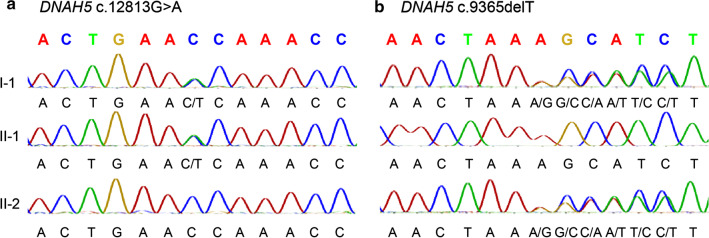
Validation of identified mutation in the pediatric patient and the parents. **a** Sanger sequencing of *DNAH5* reveals a heterozygous C > T mutation in the pediatric patient and the father. **b** Sanger sequencing of *DNAH5* reveals a heterozygous deletion of T mutation in the pediatric patient and the mother. I-1, II-1 and II-2 indicate the pediatric patient, the father and the mother individually
- *Therapy* Azithromycin was given through intravenous injection to treat M.Pneumonia, and Cefaclor Oral Suspension was given to treat Haemophilus influenzae (Hin). Acetylcysteine solution for inhalation and bromhexine hydrochloride through intravenous injection were given for relieving cough and getting rid of phlegm. Budesonide inhalation solution was given for anti-inflammatory treatment. Postural drainage and percussion, controlled coughing and active cycle of breathing techniques were performed to clear mucus from the lung. Exercise therapy was used for pulmonary rehabilitation.

## Literature review

We searched PubMed and CNKI for evidence relating to KS until Feb. 2020 using the search term “Kartagener syndrome”, “children”, “child”, “whole exome sequencing”, “DNAH5”, “primary ciliary dyskinesia (PCD)” and “situs inversus”. We further filtered the articles by limiting the age to less than 18 years as we were only looking for pediatric patients who were diagnosed with KS that was caused by mutations in *DNAH5*. 21 pediatric patients from six countries including China, the United States, the Czech Republic, Japan, Portugal and Italy, were identified among nine publications. These pediatric patients ranged from 0 to 15 years with an average age of 6.52 years. Fourteen of these cases had compound heterozygous mutations in *DNAH5*. Thirty-five nonsense, missense and frameshift mutations were identified in the 21 cases and 24 of them were novel at the time of publication. TEM was done for 15 cases, in which 11 cases (73.3%) showed outer dynein arm (ODA) defects and 4 cases (26.7) showed both ODA and inner dynein arm (IDA) defects. Six of the publications were multi-case studies. Boaretto et al. reported 51 Italian cases with PCD and 20 (39%) of them exhibited situs inversus. Genetic tests were performed for 24 of these cases. Eight of them had *DNAH5* mutations but only 1 of the 8 had KS. Ferkol et al. reported 19 American cases with PCD and 4 (21%) of them exhibited situs inversus. Twelve cases had DNAH5 mutations and 1 of them had KS. K. Takeuchi et al. reported 46 cases and 2 (4.3%) of them had situs inversus. Ten cases had mutations in PCD related genes. Seven of them were in DNAH5, but only 2 had KS. Kim et al. reported 37 cases with PCD and 16 (43.2%) of them exhibited situs inversus. Genetic tests were done for 27 of the cases. Fourteen of them had mutations in DNAH5 and 7 of the 14 cases had KS. Djakow et al. reported 21 cases with PCD and 15 (48.4%) of them exhibited situs inversus. Genetic tests were performed for 27 of these cases. Seven cases had mutations in *DNAH5* and 6 of the 7 cases had KS. Many of these pediatric patients had typical clinical manifestations of PCD together with situs inversus. However, siblings of these patients could show only symptoms of PCD without situs inversus, indicating that certain KS-causing mutations may result indifferent clinical manifestations [2–10] (Additional file 1: Table S1).

## Discussion and conclusion

KS is a subtype of PCD, an inherited disorder caused by abnormal structures and functions of cilia. In addition to the typical clinical manifestations of PCD, including chronic bronchitis, chronic rhinosinusitis, chronic otitis media, bronchiectasis and infertility, KS patients also exhibit situs inversus which accounts for 50% of PCD cases [11, 12]. There is currently a lack of epidemiological data of PCD in China, and the incidence of PCD in different populations varies markedly. The worldwide incidence of PCD is about 1/15,000–1/40,000 based on multiple studies in the past two decades [13–16]. The symptoms of PCD are non-specific, overlapping with other common respiratory conditions, therefore the actual incidence of it is probably underestimated. PCD is also an underdiagnosed disease with a common delay in diagnosis due to challenges in the diagnosis process such as lack of diagnostic services and expertise on interpreting the test results. One study has reported an average age at diagnosis of PCD as 4.4 years [17]. Analysis of 1,009 PCD patients from 26 European countries found a median age at diagnosis as 5.3 years, lower in children with situs inversus compared to those without [14]. Our literature review of 21 pediatric patients with KS showed an average age at diagnosis as 6.52 years. The average age at diagnosis of Chinese children with PCD has been determined to be 9.16 ± 3.67 years [18], and the patient in our current study was within this range. However, 73% of pediatric patients with PCD may have neonatal respiratory symptoms [19], and this percentage was 47.6% for pediatric patients with KS in our literature review. Although its clinical manifestations can appear in the neonatal period, KS is normally not a lethal condition. Therefore, pediatric patients with PCD can maintain a good quality of life and a normal survival time with early detection, regular follow-ups, prevention and treatment of complications. Early diagnosis thus has great clinical significance.

KS can be diagnosed by the presence of the clinical triad of bronchiectasis, sinusitis and situs inversus or a PCD diagnosis combined with additional situs inversus. Its clinical symptoms are often similar to other respiratory conditions, so chest imaging and nasal NO concentration can only be used as screening tests. The atypical symptoms and limitations of diagnostic tests underlie the diagnostic uncertainty. Traditionally, the detection of ultrastructural defects in cilia by TEM serves as the gold standard for the diagnosis of PCD. Ciliary beat frequency and pattern demonstrated by HSVMA is also greatly helpful in the diagnosis [20]. However, these approaches are limited by the poor compliance of tissue sampling by pediatric patients and potential secondary changes to the mucosa and the ciliary ultrastructure [12, 21]. Genetic testing is now becoming a novel diagnostic tool for PCD.

Our patient showed the clinical triad of KS together with reduced nasal NO concentration and hearing loss. Further TEM analysis of bronchial mucosal biopsy confirmed a diagnosis of KS/PCD. Besides microtubular disorganization and partial or complete loss of outer dynein arms, formation of gigantic cilia with unclear axonemal structures through fusion of multiple cilia was also observed (Fig. 1e, f).

We further ruled out the possibility that these observations were caused imaging problems and found that about 83% of the clearly captured microtubules showed absence of outer dynein arms. Immature microtubular doublets with internal disorganization were also identified in the TEM results which have been previously reported in patients with PCD previously (Fig. 1e). To confirm the diagnosis of KS, whole-exome sequencing was performed for the patient and her parents. Two compound heterozygous mutations in *DNAH5* were identified in the patient which were inherited from the parents. p. Trp4271Term was absent in the gnomAD database and p. Leu3122Term had an allele frequency of 3/250826 without homozygotes of this allele. Both mutations had not been reported before and were predicted to be pathogenic as they both result in premature stop codons. In our study, HSVMA was not performed due to inaccessibility of the equipment. Thus, a direct analysis of ciliary motility was not available in the diagnosis of the disease. However, the presence of clinical triad of KS, reduced nasal NO concentration, abnormal ciliary ultrastructure under TEM and identification of pathogenic *DNAH5* mutations in our patient all supported a diagnosis of KS.

Targeted gene sequencing panels for exons and exon–intron junctions of PCD/KS related genes are increasingly used in the final diagnosis of the disease. More than 40 PCD related genes have been identified to date [6], in which *DNAH5*, *DNAI1*, *DNAI2*, *DNAH11*, *ARMC4 and TXNDC3* cause KS [18]. These genes encode different protein components of the cilium and thus deficiency in these proteins leads to various types of pathological changes of cilia. *DNAH5* encodes the dynein axonemal heavy chain 5 protein, which is a component of the outer dynein arm. *DNAH5* is most frequently mutated in patients with PCD or KS. Homozygous or compound heterozygous mutations in *DNAH5* result in ODA defects and abnormal cilia mobility [22]. DNAH5 protein is also involved in the randomization of left–right asymmetry during embryogenesis, so patients with mutations in *DNAH5* may show situs inversus [2, 23]. In our literature review, 39–48.4% of pediatric patients with PCD also had a diagnosis with KS or SI, slightly lower than the reported 50%. Pediatric patients with mutations in *DNAH5* accounted for 15.2–51.9% of all cases with available genetic test results. The high variation in this percentage was likely due to diversity in ethnic groups. The proportion of pediatric KS patients with mutations in *DNAH5* was even lower with a percentage of only 2.3–25.9%, suggesting a lack of diagnosis in pediatric patients. Sixty-nine percent of reported *DNAH5* mutations in our literature review were novel, but their mutation types were not documented completely. Most of the pediatric patients with *DNAH5* mutations showed only ODA defects and a small portion of them showed mixed defects in ciliary ultrastructure, probably caused by additional mutations in other PCD related genes. The articles involved in our literature review had different standards for reporting mutations without including all the basic information, such as the exon number, nucleotide change, amino acid change, mutation type and zygosity. Therefore, certain information of those reported mutations is missing in our summary.

In summary, nasal NO concentration is commonly used as the screening test for diagnosis of KS. A combination of TEM and genetic testing should be used for the final diagnosis of KS. Our literature review provides a summary of the *DNAH5* mutations and ethnic distribution of pediatric KS patients, which can be used as a reference for the diagnosis of KS for pediatric patients.

## Supplementary Information

**Additional file 1.** Summary of pediatric patients with DNAH5 related Kartagener syndrome in recent literature.

**Additional file 2. Figure S2**. Quantification of ultrastructural defects in cilia by TEM. Percentage of microtubules with absence of outer dynein arms in clearly observed axonemes. Around 100 cilia of 5 cross sections were used in this calculation. Error bar indicates SEM.

## Data Availability

The datasets generated and/or analyzed during the current study are available in the [NCBI] repository, [https://www.ncbi.nlm.nih.gov/sra/?term=SRR14861240].

## Abbreviations

KS: Kartagener syndrome
PCD: Primary ciliary dyskinesia
CT: Computed tomography
TEM: Transmission electron microscopy
HSVMA: High speed video microscopy analysis
CBC: Complete blood count
PCT: Procalcitonin
CRP: C-reactive protein
BAL: Bronchoalveolar lavage
Hin: Haemophilus influenzae
ppb: Parts per billion
FEV1: Forced expiratory volume in 1 s
ODA: Outer dynein arm
IDA: Inner dynein arm
