# Supplementary material for: Novel compound heterozygous mutations of DNAH5 identified in a pediatric patient with Kartagener syndrome: case report and literature review
Source: BMC Pulm Med. 2021 Aug 14;21:263. doi: 10.1186/s12890-021-01586-4 (PMC8364053; doi:10.1186/s12890-021-01586-4)
Supplement: Supplementary file 1 — Additional file 1. Summary of pediatric patients with DNAH5 related Kartagener syndrome in recent literature. [file 12890_2021_1586_MOESM1_ESM.docx]

Suppplewment trable 1

|  |  |  |  |  | ① | ② | ②/① | ③ | ④ | ④/③ | ⑤ | ⑤/③ | ⑥ | ⑥/③ |  |  |  |  |  |  |  |  |  |  |  |
| --- | --- | --- | --- | --- | --- | --- | --- | --- | --- | --- | --- | --- | --- | --- | --- | --- | --- | --- | --- | --- | --- | --- | --- | --- | --- |
|  | Article | Authors | Year of publication | Nationality | # of PCD patients | # of SI patients | SI/PCD | # of patients | # of patients with *DNAH5* mutations | % of patients with *DNAH5* mutations | # of pediatric patients with *DNAH5* mutations | % of pediatric patients with *DNAH5* mutations | # of selected cases |  | Gender | Age |  | Exon | Nucleotide sequence change | Amino acid change | Novelty | Zygosity | Mutation type | Ultrastructual defect | NRD |
| 1 | Congenital Heart Disease and Other Heterotaxic Defects in a Large Cohort of Patients With Primary Ciliary Dyskinesia | Kennedy et al | 2007 | United States | 337 | 161 | 47.8% |  |  |  |  |  | 1 |  | F | 5 |  |  |  |  |  | Homozygous |  | ODA+IDA | Y |
| 2 | Effectiveness of Sequencing Selected Exons of DNAH5 and DNAI1 in Diagnosis of Primary Ciliary Dyskinesia | Djakow et al. | 2012 | Czech Republic | 31 | 15 | 48.4% | 27 | 7 | 25.9% | 7 | 25.9% | 6 | 22.2% |  | 4 |  | M50 | c.8396G>C | R2799P | Novel | Compound heterozygous | missense | ODA | Y |
|  |  |  |  |  |  |  |  |  |  |  |  |  |  |  |  |  |  | P61 | c.10426C>T | Q3462X | Novel |  | nonsense |  |  |
|  |  |  |  |  |  |  |  |  |  |  |  |  |  |  |  | 14 |  | 44 | c.7429C>T | Q2463X | Novel | Compound heterozygous | nonsense | ODA+IDA | N |
|  |  |  |  |  |  |  |  |  |  |  |  |  |  |  |  |  |  | 63 | c.10815delT | P3606HfsX23 | Reported |  | frameshift |  |  |
|  |  |  |  |  |  |  |  |  |  |  |  |  |  |  |  | 15 |  | 30 | c.4879C>T | Q1613X | Novel | Compound heterozygous | nonsense | ODA | N/A |
|  |  |  |  |  |  |  |  |  |  |  |  |  |  |  |  |  |  | 31 | c.5156+1G>C (IVS31+1G>C) | Splicing | Novel |  | splice site |  |  |
|  |  |  |  |  |  |  |  |  |  |  |  |  |  |  |  | 5 |  | 68 | c.11625C>A | S3861R | Novel | Heterozygous | missense | ODA | N |
|  |  |  |  |  |  |  |  |  |  |  |  |  |  |  |  | 12 |  | M50 | c.8440_8447delGAACCAAA | 2814fsX1 | Reported | Compound heterozygous | frameshift | ODA | N |
|  |  |  |  |  |  |  |  |  |  |  |  |  |  |  |  |  |  | P63 | c.10574G>A | R3539H | Novel |  | missense |  |  |
|  |  |  |  |  |  |  |  |  |  |  |  |  |  |  |  | 8 |  | 32 | c.5172A>C | K1710N | Novel | Compound heterozygous | missense | N/A | N/A |
|  |  |  |  |  |  |  |  |  |  |  |  |  |  |  |  |  |  | 54 | c.9040C>T | R3000X | Novel |  | nonsense |  |  |
| 3 | Primary Ciliary Dyskinesia-Causing Mutations in Amish and Mennonite Communities | Ferkol et al. | 2013 | United States | 19 | 4 | 21.1% | 44 | 12 | 27.3% | 1 | 2.3% | 1 | 2.3% | F | 2 |  | 27 | c.4348C>T | p.Q1450X | Novel | Compound heterozygous | nonsense | ODA | Y |
|  |  |  |  |  |  |  |  |  |  |  |  |  |  |  |  |  |  | 63 | c.10815delT | p.P3606HfsX23 | Novel |  | frameshift |  |  |
| 4 | The Role of Molecular Genetic Analysis in the Diagnosis of Primary Ciliary Dyskinesia | Raymond H. Kim, David A. Hall, Ernest Cutz et al. | 2013 | United States | 37 | 16 | 43.2% | 27 | 14 | 51.9% | 7 | 25.9% | 7 | 25.9% | F | 14 |  |  | c..9460_9461insGTTTTTCT | p.Asp3154Glyfs*23 | Novel | Homozygous | frameshift | ODA | Y |
|  |  |  |  |  |  |  |  |  |  |  |  |  |  |  | M | 2.5mo |  |  | c.10815delT(39) | p.Pro3606Hisfs*23 | Reported | Compound heterozygous | frameshift | ODA | Y |
|  |  |  |  |  |  |  |  |  |  |  |  |  |  |  |  |  |  |  | c.11571-1G>A | Splicing | Novel |  | splice site |  |  |
|  |  |  |  |  |  |  |  |  |  |  |  |  |  |  | M | 11mo |  |  | c.10384C>T(42） | p.Gln3462X | Reported | Compound heterozygous | nonsense | ODA | Y |
|  |  |  |  |  |  |  |  |  |  |  |  |  |  |  |  |  |  |  | c.1333815G>A(39) | Splicing | Reported |  | splice site |  |  |
|  |  |  |  |  |  |  |  |  |  |  |  |  |  |  | M | 4 |  |  | c.13486C>T(39） | p.Arg4496X | Reported | Heterozygous | nonsense | ODA | Y |
|  |  |  |  |  |  |  |  |  |  |  |  |  |  |  | F | 1.5mo |  |  | c.7463T>C | p.Leu2488Pro | Novel | Homozygous | missense variant | N/A | Y |
|  |  |  |  |  |  |  |  |  |  |  |  |  |  |  | F | 6 |  |  | c.3037_3040delAGCG | p.Val1014Leufs*20 | Novel | Compound heterozygous | frameshift |  |  |
|  |  |  |  |  |  |  |  |  |  |  |  |  |  |  |  |  |  |  | c.10815delT(39） | p.Pro3606Hisfs*23 | Reported |  | frameshift | Inadequate | Y |
|  |  |  |  |  |  |  |  |  |  |  |  |  |  |  | M | 0mo |  |  | c.13458dupT(39） | p.Asn4487X | Reported | Compound heterozygous | nonsense | Inconclusive | Y |
|  |  |  |  |  |  |  |  |  |  |  |  |  |  |  |  |  |  |  | c.11308A>G | p.Ser3770Gly | Novel |  | missense |  |  |
| 5 | Diagnosis of Primary Ciliary Dyskinesia by a Targeted Next-Generation Sequencing Panel Molecular and Clinical Findings in Italian Patients | Boaretto et al | 2016 | Italian | 51 | 20 | 39.2% | 24 | 8 | 33.3% | 3 | 12.5% | 1 | 4.2% | M | 9 |  | Ex.77 | c.13486C>T | p.Arg4496X | Reported | Heterozygous | nonsense | ODA | N |
| 6 | Clinical and genetic analysis of a family with Kartagener syndrome caused by novel DNAH5 mutations | Xuan X et al. | 2016 | China | 2 | 1 |  | 2 | 2 |  | 2 |  | 1 |  | M | 2mo |  | M | c.13729G>A | p.R4577X | Novel | Compound heterozygous | nonsense | N/A | N/A |
|  |  |  |  |  |  |  |  |  |  |  |  |  |  |  |  |  |  | P | c.7778C>T | p.G2593E | Novel |  | missense |  |  |
| 7 | A targeted next-generation sequencing panel reveals novel mutations in Japanese patients with primary ciliary dyskinesia | K. Takeuchi et al | 2017 | Japan | 46 | 2 |  | 46 | 7 | 15.2% | 2 | 4.3% | 2 | 4.3% | F | 7 |  | P33 | c.5367delT | N1790IfsX14 | Novel | Compound heterozygous | frameshift | ODA | N/A |
|  |  |  |  |  |  |  |  |  |  |  |  |  |  |  |  |  |  | M54 | c.9018C>T | splicing | Novel |  | splice site |  |  |
|  |  |  |  |  |  |  |  |  |  |  |  |  |  |  | F | 7 |  | P45 | c.7550_7556delAGCTGCC;c.7561_7573delCCAGCGGGGCCCG | E2517GfsX52;P2521GfsX46; | Novel | Compound heterozygous | frameshift |  |  |
|  |  |  |  |  |  |  |  |  |  |  |  |  |  |  |  |  |  | M79 | c.13837delG | V4613X | Novel |  | nonsense mutation | N/A | N/A |
| 8 | Cilia ultrastructural and gene variation of primary ciliary dyskinesia:report of three cases and literatures review. | Ke Wang et al | 2018 | China | 3 | 1 |  | 3 | 1 |  | 1 |  | 1 |  | M | 10 |  | NA | c.7576_7577insG | p.Asp2526GlyfsTe |  | Heterozygous | frameshift | ODA+IDA | N/A |
|  |  |  |  |  |  |  |  |  |  |  |  |  |  |  |  |  | DNAH11 | NA | c.5192A>T | p.Glul731Val |  |  | missense |  |  |
| 9 | Clinical and Genetic Analysis of Children with Kartagener Syndrome | Rute Pereira et al | 2019 | Portugal | 2 | 2 |  | 2 | 1 |  | 1 |  | 1 |  | M | 7 |  | M | c.4530del | p.Asn1511Metfs*6 | Novel | Compound heterozygous | frameshift | ODA+IDA | N/A |
|  |  |  |  |  |  |  |  |  |  |  |  |  |  |  |  |  |  | P | c.6000C>A | p.Tyr2000X | Novel |  | nonsense |  |  |
|  |  |  |  |  |  |  |  |  |  |  |  |  |  |  |  |  |  |  |  |  |  |  |  |  |  |
|  | ODA, defect of outer dynein arms;IDA, defect of inner dynein arms;NRD,neonatal respiratory distress;Y,yes;N,no;N/A,not available |  |  |  |  |  |  |  |  |  |  |  |  |  |  |  |  |  |  |  |  |  |  |  |  |
