# Supplementary figures and images for: Novel compound heterozygous mutations of DNAH5 identified in a pediatric patient with Kartagener syndrome: case report and literature review
Source: BMC Pulm Med. 2021 Aug 14;21:263. doi: 10.1186/s12890-021-01586-4 (PMC8364053; doi:10.1186/s12890-021-01586-4)

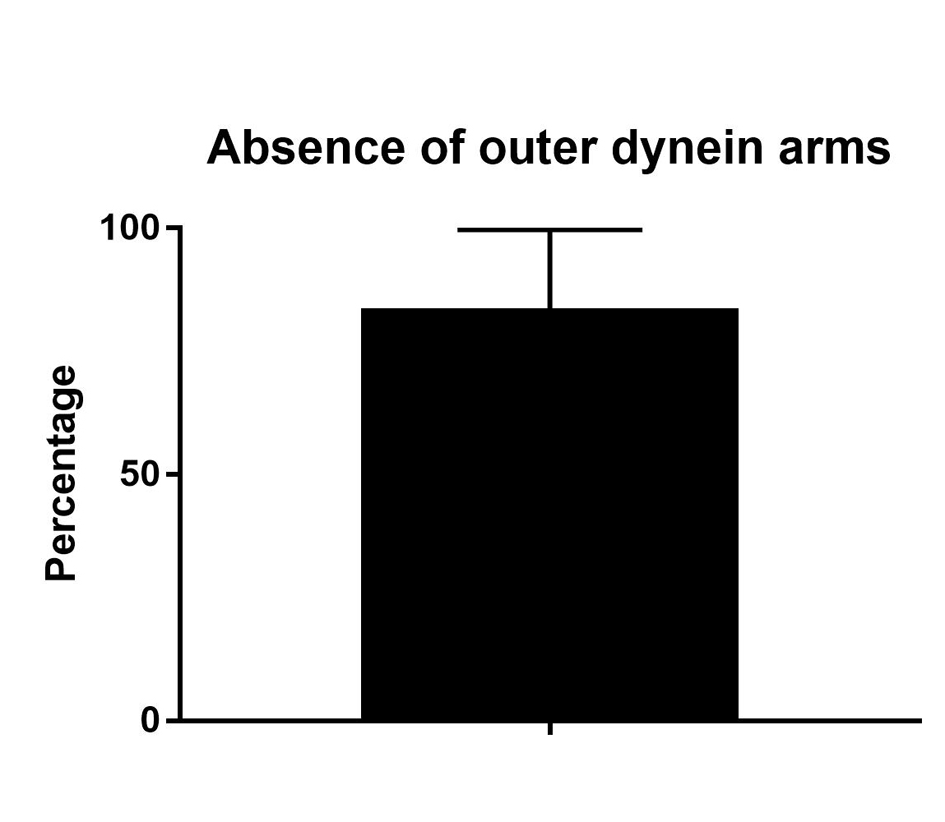

Supplement: Supplementary file 2 — Additional file 2. Figure S2. Quantification of ultrastructural defects in cilia by TEM. Percentage of microtubules with absence of outer dynein arms in clearly observed axonemes. Around 100 cilia of 5 cross sections were used in this calculation. Error bar indicates SEM. [file 12890_2021_1586_MOESM2_ESM.tif]
